# Clinical-pathological characteristics and prognostic factors for malignant peritoneal mesothelioma in the elderly

**DOI:** 10.1186/s12876-022-02361-3

**Published:** 2022-06-09

**Authors:** Dan Pan, Mengyao Wang, Wencheng Liu, Yan Li, Lixuan Sang, Bing Chang

**Affiliations:** 1 Department of Geriatrics, The First Affiliated Hospital of China Medical University, Shenyang, China; 2 Department of Gastroenterology, Shengjing Hospital of China Medical University, Shenyang, China; 3 Department of Gastroenterology, The First Affiliated Hospital of China Medical University, Shenyang, China

**Keywords:** Malignant peritoneal mesothelioma, Elderly patients, Pathological features, Prognostic factors, Cancer-specific survival

## Abstract

**Background:**

Malignant peritoneal mesothelioma (MPM) is a rare disease characterized by atypical symptoms, difficult diagnosis, variable course and poor prognosis, and it develops mainly in elderly individuals. The authors aimed to identify the clinical-pathological characteristics, prognosis, and prognostic factors in elderly MPM patients.

**Methods:**

From the National Cancer Institute Surveillance Epidemiology End Results (SEER) database, 1492 patients with MPM from 1975 to 2016 were selected and divided into the elderly group (≥ 65) and the adult group (< 65). We compared the clinical-pathological characteristics and treatment methods of the elderly group (N = 665) and the adult group (N = 827). At the same time, we analysed specific selected clinicopathological parameters and prognostic factors for elderly MPM patients.

**Results:**

Compared with the adult group, the elderly group had higher percentages of male patients (*P* = 0.017) and white patients (*P* = 0.043) and lower proportions of insured patients (*P* < 0.001) married patients (*P* < 0.001), patients with peritoneal tumours (*P* = 0.006) and patients who underwent surgery (*P* < 0.001) and chemotherapy (*P* < 0.001). There was a significant difference in the differentiation grade between the two groups (*P* = 0.003). Elderly patients had a shorter median survival time than adult patients (6 months vs. 19 months). Uninsured (hazard ratio (HR): 5.187, *P* = 0.005), sarcomatoid type (HR 3.913, *P* < 0.001), poorly differentiated (HR 3.900, *P* < 0.001), distant metastasis (HR 1.735, *P* = 0.001), no cancer-directed surgery (HR 1.733, *P* < 0.001), and no chemotherapy (HR 1.532, *P* < 0.001) were independently associated with poorer prognosis in elderly MPM patients.

**Conclusion:**

Compared with adult patients, elderly MPM patients had a higher male ratio, poor differentiation and relatively conservative treatment. The cancer-specific survival (CSS) rate of elderly MPM patients was significantly lower than that of adult patients. Insurance status, histology type, differentiation grade, stage, surgery status, and chemotherapy status were all independent prognostic factors for elderly MPM patients.

## Introduction

Malignant mesotheliomas (MM) are aggressive tumours arising from serous surfaces, including the pleura (65%-70%), peritoneum (30%), tunica vaginalis testis, and pericardium (1%-2%) [1]. Occupational or environmental exposures to asbestos are considered to be pathogenic factors [2], and the annual number of MM deaths is increasing, particularly among persons aged above 85 years old, most likely representing exposure many years ago [3]. As a subgroup of MM, malignant peritoneal mesothelioma (MPM) is a rare cancer originating from mesothelial tissues inside the patient's abdominal cavity and was first identified by Miller and Wynn in 1908 [4]. Radiation could also be implicated as a factor favouring the development of peritoneal mesothelioma [5]. Approximately 1–2 in 1 million people are diagnosed with MPM each year, with an annual incidence in the United States of 200–400 new cases [1]. The patients are mainly elderly, with a median diagnosis age of 64 years [6], and the incidence rate in the elderly has been increasing in recent years [7]. Previous reports have found that approximately 40–60% of patients have metastases at the time of diagnosis, and the median survival time for untreated patients is less than 1 year [1, 6, 8]. It was demonstrated that age is an important prognostic factor of MPM, and adult patients generally had a better prognosis than elderly patients, with a significantly longer median survival time [8, 9]. Moreover, sex [10–12], histology type [6, 11, 13–16], tumour stage [8, 17], differentiation grade [12], and surgery status [18, 19] were also regarded as prognostic factors for MPM, but the results varied from study to study.

However, there have been few studies on the clinical-pathological characteristics and prognostic factors of elderly patients. Therefore, in this study, we selected patients from the Surveillance, Epidemiology and End Results (SEER) database [20]. With these patients, we studied a number of selected clinical-pathological and treatment characteristics of adult and elderly patients as well as the 1-, 3- and 5-year cancer-specific survival (CSS). Then, we identified the prognostic factors that were associated with CSS in elderly MPM patients.

## Materials and methods

### Data source and study subjects

A retrospective case series analysis was performed using the SEER database. The database records patients' basic information, diagnosis basis, stage and grade, treatment plan, follow-up time and cause of death, covering approximately 28% of cancer patients in the United States, providing valuable oncology data for medical researchers worldwide.

Patients with pathologically confirmed MPM were enrolled from 1975 to 2016 using ICD-O-3 histology codes 9050-9053 (mesothelioma, malignant) combined with site codes 48.0, retroperitoneum; 48.1, specified parts of the peritoneum; 48.2, peritoneum not otherwise specified; and 48.8, overlapping lesion of retroperitoneum & peritoneum. Patients who had other malignancies or did not have active follow-up since the time of MPM diagnosis were excluded from our study cohort. Based on these criteria, our study cohort consisted of a total of 1,492 MPM patients.

Demographic variables of interest to our study included gender, race, age, marital status, and insurance status; clinical data of interest included the status of surgery, radiotherapy, chemotherapy, and survival time (from MPM diagnosis until cause-specific death (CSD), as of December 31, 2016, in months); pathological characteristics of interest included histology type, differentiation grade, and tumour stage.

For further analysis, some demographics and clinical factors were classified based on SEER database record and our clinical experiences: for insurance status, patients with “Any Medicaid”, “Insured”, and “Insured/no specifics” were regarded as the “Insured” group, for marital status, “Separated”, “Divorced”, “Single”, and “Widowed” were clustered as the “Unmarried” group, differentiated grade was defined as “Well differentiated”, “Moderately differentiated”, “Poorly differentiated”, “Undifferentiated” or “Unknown”, tumour staging was defined as “Localized”, “Regional”, “Distant”, or “Unknown”, and histological type was defined as “Epithelioid”, “Sarcomatoid”, “Biphasic” or “Unknown”, radiotherapy and chemotherapy status were defined as “Yes” or “No or unknown”, for surgery status, the database did not contain details of MPM surgery before 1987, and the classification of surgery has also changed over time since 1987, so surgery status was classified as “Cancer-directed surgery done”, “No cancer-directed surgery” or “Unknown” groups in order to obtain long-term data.

### Statistical methods

Statistical analyses were performed using SPSS software (version 26.0). The study subjects were stratified by age into two groups: the adult group (< 65) and the elderly group (≥ 65). Using Student’s t test and the chi-square (χ2) test, selected clinical-pathological and treatment characteristics were compared between the adult and elderly groups as well as between the males and females in the elderly group. The CSS rate was determined by the Kaplan–Meier method, and the differences in CSS rates were determined by the 2-sided log-rank test. Cox proportional hazards analysis was performed to assess the independent risk factors for CSD in the elderly group. All *P* values were two-sided, and *P* < 0.05 was considered to be statistically significant.

## Results

### Patient clinical-pathological and treatment characteristics

From 1975 to 2016, a total of 1492 MPM patients were identified from the SEER registry, and there were 665 individuals in the elderly group (≥ 65). The baseline characteristics of the selected patients are summarized in Table 1. Compared with the adult group, the elderly had a higher percentage of males (58.80% > 52.60%, *P* = 0.017) and white patients (92.63% > 89.12%, *P* = 0.043) and lower proportions of insured patients (35.94% < 38.21%, *P* < 0.001), married patients (59.85% < 60.78%, *P* < 0.001) and patients with peritoneum tumours (94.74% < 97.58%, *P* = 0.006). The differentiation grade in elderly patients was relatively worse than that in adult patients (*P* = 0.003), the ratios of well-differentiated patients (4.96% < 10.16%) and moderately differentiated patients (1.65% < 2.06%) were lower than those in adult patients, and the ratio of poorly differentiated patients was higher than that in adult patients (7.22% > 5.68%). Surgery (33.38% < 47.88%, *P* < 0.001) and chemotherapy (44.21% < 59.7%, *P* < 0.001) incidence was less common in elderly patients than in adult patients.

**Table 1:** Baseline characteristics of the adult and elderly MPM patients

Variable	< 65 years old^a^ N = 827 (55.43%)	≥ 65 years old^a^ N = 665 (44.57%)	*P* value
*Gender*			**0.017**
Male	274 (41.20%)	391 (58.80%)	
Female	274 (41.20%)	274 (41.20%)	
*Race*			**0.043**
White	737 (89.12%)	616 (92.63%)	
Black	47 (5.68%)	24 (3.61%)	
Asian or Pacific Islander	31 (3.75%)	23 (3.46%)	
American Indian/Alaska Native	7 (0.85%)	2 (0.30%)	
Unknown	5 (0.60%)	0	
*Insurance status*			** < 0.001**
Insured	316 (38.21%)	239 (35.94%)	
Uninsured	27 (3.26%)	3 (0.45%)	
Unknown	484 (58.52%)	423 (63.61%)	
*Marital status*			** < 0.001**
Married	527 (60.78%)	398 (59.85%)	
Unmarried	256 (38.50%)	271 (31.26%)	
Unknown	69 (7.96%)	11 (1.65%)	
*Site*			**0.006**
Peritoneum	807 (97.58%)	630 (94.74%)	
Retroperitoneum	16 (1.93%)	32 (4.81%)	
Ovarlapping lesion of retroperitoneum and peritoneum	4 (0.48%)	3 (0.45%)	
*Histology*			0.403
Epithelioid	270 (32.65%)	195 (29.32%)	
Biphasic	30 (3.63%)	19 (2.86%)	
Sarcomatoid	21 (2.54%)	19 (2.86%)	
Unknown	506 (61.19%)	432 (64.96%)	
*Grade*			**0.003**
Well differentiated	84 (10.16%)	33 (4.96%)	
Moderately differentiated	17 (2.06%)	11 (1.65%)	
Poorly differentiated	47 (5.68%)	48 (7.22%)	
Undifferentiated	18 (2.18%)	11 (1.65%)	
Unknown	661 (79.93%)	562 (84.51%)	
*Stage*			0.751
Localized	86 (10.40%)	68 (10.23%)	
Regional	128 (15.48)	95 (14.29%)	
Distant	493 (59.61%)	414 (62.26%)	
Unknown	120 (14.51%)	88 (13.23%)	
*Surgery*			** < 0.001**
Cancer-directed surgery	396 (47.88%)	220 (33.38%)	
No cancer-directed surgery	407 (49.21%)	430 (64.66%)	
Unknown	24 (2.90%)	13 (1.95%)	
*Radiotherapy*			0.100
Yes	31 (3.75%)	15 (2.26%)	
No or unknown	796 (96.25%)	650 (97.74%)	
*Chemotherapy*			** < 0.001**
Yes	494 (59.7%)	294 (44.21%)	
No or unknown	333 (40.3%)	371 (55.79%)	

Significant *P* values shown in bold

MPM, malignant peritoneal mesothelioma

^a^refers to the age recorded at the time of MPM diagnosis

### The clinical-pathological characteristics of elderly patients

There were 391 (58.80%) males and 274 (41.20%) females in the elderly group. For the elderly MPM patients, the comparison of selected clinical-pathological characteristics between males and females is shown in Table 2. Compared with female patients, most of the male patients were diagnosed with MPM at a younger age (73.68 vs. 75.28, *P* = 0.002). There was a higher percentage of males than females who underwent surgery (44.53% vs. 25.58%, *P* < 0.001).

**Table 2:** Baseline characteristics of the female and male elderly MPM patients

Variable	Male N = 391 (58.80%)	Female N = 274 (41.20%)	*P* value
Age at diagnosis	73.68 ± 6.21	75.28 ± 7.06	**0.002**
*Race*			0.083
White	369 (94.37%)	247 (90.15%)	
Black	10 (2.56%)	14 (5.11%)	
Asian or Pacific Islander	12 (3.07%)	11 (4.01%)	
American Indian/Alaska Native	0	2 (0.73%)	
*Insurance status*			0.838
Insured	137 (35.04%)	102 (37.23%)	
Uninsured	2 (0.51%)	1 (0.36%)	
Unknown	252 (64.45%)	171 (62.41%)	
*Marital status*			** < 0.001**
Married	287 (73.40%)	111 (40.51%)	
Unmarried	99 (25.32%)	157 (57.30)	
Unknown	5 (1.28%)	6 (2.19%)	
*Site*			0.878
Peritoneum	369 (94.37%)	261 (95.26%)	
Retroperitoneum	20 (5.12%)	12 (4.38%)	
Ovarlapping lesion of retroperitoneum and peritoneum	2 (0.51%)	1 (0.36%)	
*Histology*			0.531
Epithelioid	116 (29.67%)	79 (28.83%)	
Biphasic	11 (2.81%)	8 (2.92%)	
Sarcomatoid	8 (2.05%)	11 (4.01%)	
Unknown	256 (65.47%)	176 (64.23%)	
*Grade*			0.274
Well differentiated	14 (3.58%)	19 (6.93%)	
Moderately differentiated	7 (1.79%)	4 (1.46%)	
Poorly differentiated	27 (6.91%)	21 (7.66%)	
Undifferentiated	5 (1.28%)	6 (2.19%)	
Unknown	338 (86.44%)	224 (81.76%)	
*Stage*			0.190
Localized	32 (8.18%)	36 (13.14%)	
Regional	58 (14.83%)	37 (13.50%)	
Distant	251 (64.19%)	163 (59.49%)	
Unknown	50 (12.79)	38 (19.87%)	
*Surgery*			** < 0.001**
Cancer-directed surgery done	100 (25.58%)	122 (44.53%)	
No cancer-directed surgery	284 (72.63%)	146 (53.28%)	
*Radiotherapy*			0.605
Yes	10 (2.56%)	5 (1.82%)	
No/unknown	381 (97.44%)	269 (98.18%)	
*Chemotherapy*			0.342
Yes	178 (45.78%)	115 (42.97%)	
No/unknown	212 (54.22%)	159 (58.03%)	

Significant *P* values shown in bold

MPM, malignant peritoneal mesothelioma

### Survival differences

**Fig. 1:**
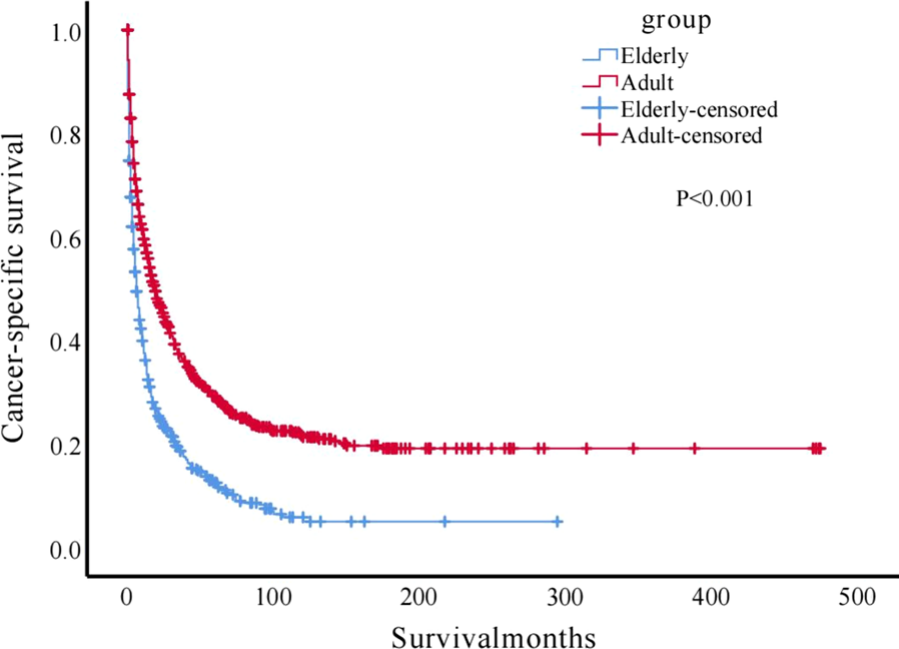
Kaplan–Meier survival analysis between elderly and adult MPM patients

As of December 31, 2016, a total of 549 patients in the elderly group (N = 665) had died. The median survival time was six months, and the 1-, 3- and 5-year CSS rates in the adult and elderly patients are shown in Table 3. The elderly patients had a significantly poorer survival than the adult patients (*P* < 0.001, Fig. 1).

**Table 3:** Comparison of the 1-, 3- and 5-year CSS rate between adult and elderly MPM patients

	Number	Death toll	Median survival time(months)	1-year CSS rate (%)	3-year CSS rate (%)	5-year CSS rate (%)
< 65^a^	827	569	19	58.41	36.96	29.30
≥ 65^a^	665	549	6	36.18	18.68	12.53
*P* value^b^				** < 0.001**	** < 0.001**	** < 0.001**

Significant *P* values shown in bold

MPM, malignant peritoneal mesothelioma; CSS, cancer-specific survival

^a^refers to the age recorded at the time of MPM diagnosis; ^b^ refers to the comparison of cause specific survival rate

The median survival time and the 1-, 3- and 5-year CSS rates in the different groups of elderly MPM patients are shown in Table 4. The 1-, 3- and 5-year CSS rates of male patients were 31.46%, 15.73%, and 10.88%, respectively, which were lower than those of female patients. The patients with distant metastasis had the lowest CSS rate compared with other groups in the same category (*P* < 0.001). The patients who had surgery for MPM had a higher 5-CSS rate than those who did not (19.14% vs. 8.76%).

**Table 4:** The influence of specific prognostic factors on the CSS of elderly MPM patients, based on the log-rank test

Variable	Number	Death toll	Median survival time (months)	1-year CSS rate (%)	3-year CSS rate (%)	5-year CSS rate (%)	*P* value (CSS)^a^
Gender							**0.006**
Male	391	335	5	31.46	15.73	10.88	
Female	274	214	9	43.1	22.97	14.79	
Race							0.507
White	616	511	6	36.27	18.07	1175	
black	24	16	8	–	–	–	
Asian or Pacific Islander	23	21	2	–	–	–	
American Indian/Alaska Native	2	1	7	–	–	–	
Insurance status							** < 0.001**
Insured	239	167	10	45.77	27.82	20.86	
Uninsured	3	3	1	–	–	–	
Unknown	423	379	5	31.20	14.23	8.75	
Marital status							**0.046**
Married	398	337	6	33.8	16.08	10.55	
Unmarried	256	205	7	38.77	21.47	13.99	
Unknown	11	7	15	–	–	–	
Site							0.936
Peritoneum	630	522	6	36.41	18.54	12.52	
Retroperitoneum	32	25	3	–	–	–	
Ovarlapping lesion of Retroperitoneum &peritoneum	3	2	1	–	–	–	
Histology							** < 0.001**
Epithelioid	195	147	13	50.77	26.41	19.69	
Biphasic	19	17	4	–	–	–	
Sarcomatoid	19	18	1	–	–	–	
Unknown	432	341	5	32.06	16.87	10.44	
Grade							** < 0.001**
Well differentiated	33	17	51	–	–	–	
Moderately differentiated	11	7	7	–	–	–	
Poorly differentiated	48	46	3	–	–	–	
Undifferentiated	11	9	4	–	–	–	
Unknown	562	470	6	36.08	18.27	12.31	
Stage							** < 0.001**
Localized	68	43	16	57.98	31.37	22.75	
Regional	95	78	11	46.13	27.58	18.47	
Distant	414	356	5	29.72	12.85	8.38	
Unknown	88	72	6	39.46	26.03	17.36	
Surgery							** < 0.001**
Cancer-directed surgery done	222	170	13	53.47	28.65	19.14	
No cancer-directed surgery	430	370	4	26.65	12.96	8.76	
Unknown	13	9	16	–	–	–	
Radiotherapy							0.173
Yes	15	15	6	–	–	–	
No/Unknown	650	534	6	36.8	19.19	12.88	
Chemotherapy							**0.001**
Yes	294	240	10	43.11	21.99	14.25	
No/Unknown	371	309	3	30.72	16.08	11.25	

Significant *P* values shown in bold

MPM, malignant peritoneal mesothelioma; CSS, cancer-specific survival

^a^refers to the comparison of overall CSS until the end of follow up; “–” refers that the number of people in the corresponding group was less than 50 and survival rates were not counted

### Univariate and multivariate analyses of risk factors for CSS rates of elderly MPM patients

**Fig. 2:**
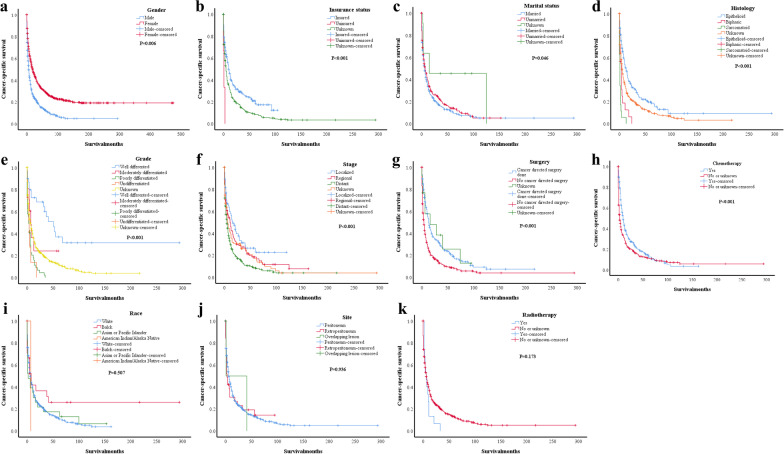
Kaplan–Meier survival analysis of CSS between different groups in elderly MPM patients: **a** between different gender groups (*P* = 0.006); **b** between different insurance status groups (*P* = 0.001); **c** between different marital status groups (*P* = 0.046); **d** between different histology type groups (*P* < 0.001); **e** between different differentiation grade groups (*P* < 0.001); **f** between different tumor stage groups (*P* < 0.001); **g** between different surgery status groups (*P* < 0.001); **h** between different chemotherapy status groups (*P* < 0.001); **i** between different race groups (*P* = 0.507);** j** between different lesion sites groups (*P* = 0.936); **k** between different radiotherapy status groups (*P* = 0.173)

Factors that were associated with the CSS of elderly patients included gender (*P* = 0.006, Fig. 2a), insurance status (*P* = 0.001, Fig. 2b), marital status (*P* = 0.046, Fig. 2c), histology type (*P* < 0.001, Fig. 2d), differentiation grade (*P* < 0.001, Fig. 2e), tumour stage (*P* < 0.001, Fig. 2f), surgery status (*P* < 0.001, Fig. 2g) and chemotherapy status (*P* < 0.001, Fig. 2h). Race (Fig. 2i), lesion site (Fig. 2j), and radiotherapy status (Fig. 2k) were not associated with the CSS of elderly patients according to our study.

**Fig. 3:**
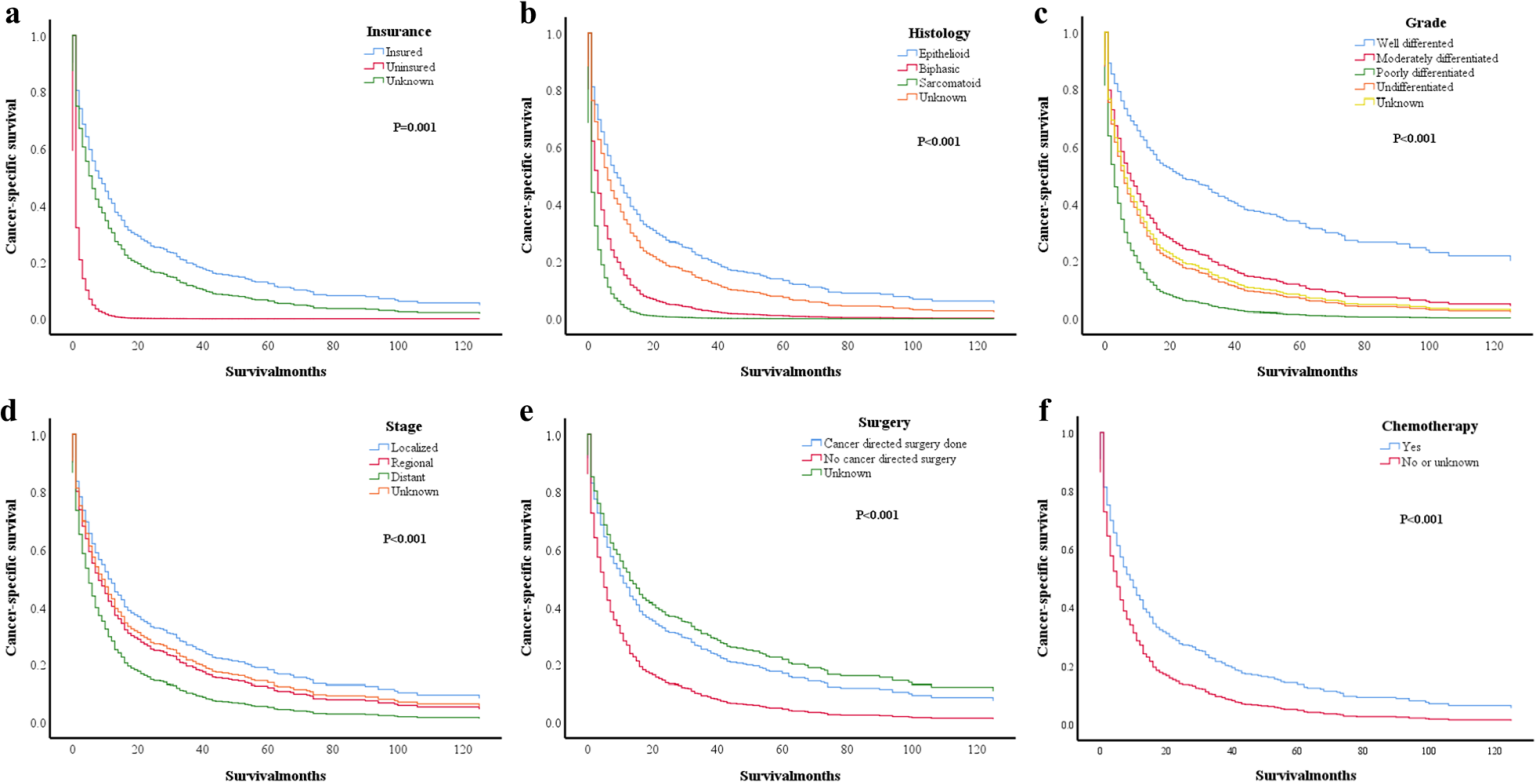
Cox proportional hazards analysis of CSS between different groups in the elderly MPM groups: **a** between different insurance status groups (*P* = 0.001); **b** between different histology type groups (*P* < 0.001); **c** between different differentiation grade groups (*P* < 0.001); **d** between different tumor stage groups (P < 0.001); **e** between different surgery status groups (*P* < 0.001); **f** between different chemotherapy status groups (*P* < 0.001)

These associated variables were included in the multivariate analysis. Cox proportional hazards analysis showed that insurance status (*P* = 0.001, Fig. 3a), histology type (*P* < 0.001, Fig. 3b), differentiation grade (*P* < 0.001, Fig. 3c), tumour stage (*P* < 0.001, Fig. 3d), surgery status (*P* < 0.001, Fig. 3e), and chemotherapy status (*P* < 0.001, Fig. 3f) were all independent prognostic factors for elderly MPM patients. As shown in Table 5, compared with insured patients, the uninsured group had a higher risk of developing CSD (hazard ratio (HR): 5.187, *P* = 0.005). Notably, elderly MPM patients with the biphasic and sarcomatoid types had a higher risk of CSD than those with the epithelioid type (biphasic type, HR 2.279, *P* = 0.002; sarcomatoid type, HR 3.913, *P* < 0.001). The poorly differentiated and undifferentiated patients had a lower CSS rate (poorly differentiated, HR 3.900, *P* < 0.001; undifferentiated, HR 2.430, *P* = 0.041) than well differentiated patients. Distant metastasis was a risk factor for poor prognosis (HR 1.735, *P* = 0.001). The no surgery group had 1.733 times the CSD risk of the surgery group. Moreover, patients in the no chemotherapy or unknown group had a 53.2% higher risk than those who underwent chemotherapy.

**Table 5:** Univariate and multivariate analysis of the elderly MPM patients

Variable	Univariate analysis		Multivariate analysis	
	Log-rank χ^2^ test	*P*-value	HR (95% CI)	*P* value
*Insurance status*	26.663	< 0.001		
Insured			Reference	
Uninsured			5.187 (1.628–16.524)	**0.005**
Unknown			1.326 (1.100–1.600)	**0.003**
*Histology*	58.350	< 0.001		
Epithelioid			Reference	
Biphasic			2.279 (1.339–3.877)	**0.002**
Sarcomatoid			3.913 (2.347–6.523)	** < 0.001**
Unknown			2.301 (1.400–3.782)	**0.012**
*Grade*	39.596	< 0.001		
Well differentiated			Reference	
Moderately differentiated			1.978 (0.811–4.824)	0.134
Poorly differentiated			3.900 (2.194–6.933)	** < 0.001**
Undifferentiated			2.430 (1.038–5.689)	**0.041**
Unknown			2.301 (1.400–3.782)	**0.001**
*Stage*	23.936	< 0.001		
Localized			Reference	
Regional			1.244 (0.855–1.811)	0.254
Distant			1.735 (1.255–2.401)	**0.001**
Unknown			1.165 (0.792–1.713)	0.438
*Surgery*	43.605	< 0.001		
Cancer-directed surgery			Reference	
No cancer-directed surgery			1.733 (1.433–2.095)	** < 0.001**
Unknown			0.857 (0.435–1.689)	0.656
*Chemotherapy*	18.157	< 0.001		
Yes			Reference	
No or unknown			1.532 (1.282–1.831)	** < 0.001**

Significant *P* values shown in bold

MPM, malignant peritoneal mesothelioma; HR, hazard ratio; CI, confidential interval

## Discussion

MPM is a rare, aggressive tumour regarded as a universally fatal disease. Despite the implementation of regulatory actions and the reduction in asbestos use, the annual number of MPM deaths remains substantial [7, 8, 21]. The elderly make up the majority of the patients, and it has been shown that elderly patients have a poorer prognosis than adult patients [8, 9, 13]. Our study utilized the SEER database to conduct an in-depth analysis of elderly MPM patients.

Among our study population, the number of male patients was higher than that of females, similar to the results of previous studies. To explain such a sex difference, it has been proposed that compared with women, men have more occupational exposure to asbestos, thereby leading to a higher incidence of MPM in males [22, 23]. In contrast to previous studies, in our study, there was a larger proportion of males among the elderly patients than in the adult group (58.80% vs. 52.60%), while a multinational, multicentre study published in 2011 suggested a prominently higher proportion of men aged 55 and below than patients over 55 (59% vs. 41%) [24]. The reason may be that all cases in this study were taken from the SEER database, which is only a collection of data from 18 regions in the U.S. mainland, and there are some regional limitations to the results.

Due to the low incidence and the shortage of reports based on large-sample studies in various regions, there are few data on survival analysis of MPM patients; moreover, the results of different regional studies are inconsistent, but the general survival time without treatment is less than 1 year. Salo Sas et al. [25] reported a median survival of only 4 months for 90 MPM patients in Finland between 2000 and 2012. John T. Miura et al. [6] suggested a median overall survival of 9 months, and V. de Pangher Manzini et al. [26] showed an even longer survival of 13 months. Regarding the survival comparison between elderly and adult patients, the elderly were found to be associated with worse survival [10, 13]. However, Cao C et al. showed that there was no significant difference in the survival time between male MPM patients older than 55 and younger than 55 [25]. In this study, elderly patients showed a shorter median survival time than adults (6 months vs. 19 months), and the survival rate was significantly lower than that of adults. This may be because elderly individuals generally have weaker health, more age-dependent physiological changes, and more complications than adults and tend to receive palliative treatment.

It was found that females with MPM generally had better outcomes than males, without considering age, time to diagnosis, and histology type [11, 24]. A 2018 case study showed that female patients had a higher 5-year survival rate than male patients (33% > 12%) [13]. Similarly, in this study, the median survival time of female MPM patients in the elderly group was 9 months, which was longer than that of males (5 months). Further analysis revealed that only 25.58% of elderly male patients underwent surgery, while 44.53% of female patients did, suggesting that females can receive more aggressive and effective treatment than males.

Several tumour-related studies suggested that the prognosis of married patients was better than that of single patients because of earlier disease detection, better financial support and more health care resources [27–29]. Contrary to our expectation, in this study, married patients had a shorter median survival time and slightly lower CSS than unmarried patients. After further analysis, we found that the majority of unmarried patients were female (61.34%) in our study, and females with MPM generally had better outcomes. Considering that male accounted for most of the married patients (72.11%), we may be able to explain why married patients’ prognosis was worse in our study.

MPM can be divided into epithelial, sarcomatoid and biphasic types based on histology, with the majority of patients having the epithelioid type [8]. Several studies have suggested that the prognosis of MPM is related to histological type. Yan et al. [18] found that patients with epithelioid MPM had a median survival of 63 months, compared with 16 months for patients with the sarcomatoid or biphasic type. Our study showed that the median survival time of patients with epithelial MPM was 13 months, compared with 4 months for patients with the biphasic type and 1 month for patients with the sarcomatoid type. In this study, the median survival time of each group was relatively short, which may be because Yan et al. included young people, and the prognosis of young patients was better than that of elderly patients; thus, the survival times would be different. In our study, the epithelial type accounted for a significantly higher proportion than the sarcomatoid and biphasic types in both the young group and the elderly group, suggesting that the distribution of histology was unrelated to age. Multivariate analysis showed that there were significant differences in CSS among patients with different pathological types, and the levels of risk of CSD in patients with biphasic and sarcomatoid MPM were 2.279 and 3.913 times higher than that in patients with the epithelioid type, respectively. Shavelle et al. [8] performed a retrospective analysis of 1229 MPM patients aged 40 or older and concluded that patients with the sarcomatoid type had a 117% higher risk than those with the epithelioid type, and patients with the biphasic type had a 44% higher risk than patients with the epithelial type. Large-scale multicentre studies at the international level will be needed in the future to verify the relationship.

Regarding the relationship between differentiation and prognosis in elderly MPM patients, consistent with previous research, our study found that patients with high differentiation had a better prognosis than others. The median survival time of patients with high differentiation was 51 months, which was much longer than that of patients with moderately differentiated, poorly differentiated and undifferentiated tumours (7 months, 3 months, 4 months). A study published in 2009 [12] concluded that patients with well-differentiated tumours had a superior median survival time when compared to those with moderately, poorly, and undifferentiated tumour grades (88 months vs. 14 months vs. 6 months vs. 6 months, respectively). The levels of risk of CSD in moderately differentiated, poorly differentiated and undifferentiated patients were 0.978, 2.900 and 1.430 times higher than that in well-differentiated patients, respectively.

At present, there is no mature TNM staging system for MPM. Yan et al. proposed a TNM staging system for diffuse malignant peritoneal mesothelioma in 2010 [30]. The SEER database was used to divide the patients into a localized staging group, a regional staging group and a distant metastasis group. It was shown that 40–60% of MPM patients had distant metastasis at the time of detection [6, 8], consistent with our finding (50%). Our results showed that the 5-year CSS rates of elderly MPM patients in localized, regional, and distant stages were 22.75%, 18.47% and 8.38%, respectively. Distant metastasis was independently associated with poor survival.

For treatment, the effect of radiotherapy for MPM patients is not clear. Silja A.S. Salo et al. [19] showed that for patients who were treated with radiotherapy alone, the median survival time was 2 months, and the 1-year CSS rate was 20%. Our study shows that patients who had radiotherapy or not (including the unknown group) had the same median survival time of 6 months, and the results showed that radiation therapy had no obvious effect on the prognosis of elderly MPM patients.

In this study, only 33.38% of all elderly MPM patients underwent surgery, consistent with Anish Thomas’ report in 2015 (32%) [10]. Surgical interventions have been proven to be associated with better outcomes [12]. We observed that the 5-year survival rate of the patients who underwent surgery was higher than that of those who did not (19.14% vs. 8.76%). The no-surgery group had 1.733 times the CSD risk of the surgery group, and surgery was the treatment option to improve prognosis. However, elderly patients mostly have multiple and complex underlying diseases and tend to receive palliative treatment.

Chemotherapy is often combined with surgery to treat MPM, which can be delivered in the form of heated intraperitoneal chemotherapy (HIPEC). Nagata Y et al. [31] found that cisplatin plus pemetrexed showed consistent efficacy with MPM and can be recommended as a first-line treatment for unresectable MPM. Yan et al. [18] reported a median survival of 56 months for 372 patients who received HIPEC and 23 months for those who did not (*P* = 0. 049). However, some studies have shown that systemic chemotherapy has no positive effect on the prognosis of MPM patients [32]. In this study, patients receiving chemotherapy showed a longer median survival time than those who did not receive it or were unsure (13 months vs. 10 months). Approximately 44.21% of elderly patients underwent chemotherapy, far more than surgery or radiotherapy. Multivariate analysis demonstrated that chemotherapy was independently associated with improved survival outcomes.

At present, cytoreductive surgery (CRS) combined with HIPEC as the first-line treatment of MPM has been proven to improve the prognosis of MPM patients [33–37]. A systematic review and meta-analysis showed that patients receiving CRS and HIPEC had a median survival time of 29.5–100 months, much longer than that of untreated patients [38]. CRS surgery is suitable for patients below 75 years of age without distant metastasis and with no contraindication signs of operation [39]. However, there are few elderly patients who can meet the above conditions. In addition, Deepa Magge et al. [16] found that there may be no benefit gained from CRS-HIPEC in the sarcomatoid type and biphasic groups compared to those with the epithelioid type. Thus, more clinical studies on elderly MPM patients are necessary.

### Limitations of this study

However, several limitations in our study should be considered. First, the SEER database does not clearly distinguish patients who did not receive chemotherapy or radiotherapy and those who did not know whether they had received these treatments, and we could not determine the effect of chemotherapy or radiotherapy on elderly MPM patients more precisely. Second, there are no specific chemotherapy regimens in SEER, so the influence of different chemotherapy regimens on prognosis cannot be studied. Third, for the variables of histology type and differentiation grade, the majority of patients were in the unknown group, which affected the accuracy of our results.

## Conclusion

In conclusion, for elderly MPM patients, insured, epithelioid type, and well differentiated are favourable prognostic factors; while distant metastasis and no surgery or chemotherapy are independently associated with poorer prognosis. Moreover, the elderly generally have weaker health, more age-dependent physiological changes and palliative treatments. As such, to provide effective treatment and extend the lifespan of elderly MPM patients, all risk factors and the specific conditions of patients must be carefully assessed in the determination of treatment strategies.

## Data Availability

The datasets analysed in the current study are available in the Surveillance, Epidemiology and End Results (SEER) database (https://seer.cancer.gov/data/).

## Abbreviations

MPM: Malignant peritoneal mesothelioma
MM: Malignant mesothelioma
CSS: Cancer-specific survival
CSD: Cause-specific death
HR: Hazard ratio
CRS: Cytoreductive surgery
HIPEC: Heated intraperitoneal chemotherapy
